# Draft Genome Sequence of Geotoga petraea Strain HO-Geo1, Isolated from a Petroleum Reservoir in Russia

**DOI:** 10.1128/MRA.00706-19

**Published:** 2019-07-18

**Authors:** Denis S. Grouzdev, Ekaterina M. Semenova, Diyana S. Sokolova, Tatiyana P. Tourova, Andrey B. Poltaraus, Tamara N. Nazina

**Affiliations:** a Institute of Bioengineering, Research Center of Biotechnology, Russian Academy of Sciences, Moscow, Russian Federation; b Winogradsky Institute of Microbiology, Research Center of Biotechnology, Russian Academy of Sciences, Moscow, Russian Federation; c Engelhardt Institute of Molecular Biology, Russian Academy of Sciences, Moscow, Russian Federation; University of Southern California

## Abstract

The draft genome sequence of Geotoga petraea strain HO-Geo1, a bacterium isolated from production water of the Vostochno-Anzirskoe petroleum reservoir in Russia, is presented. The genome of strain HO-Geo1 is annotated for elucidation of the metabolic potential and its possible function in the subsurface microbial community and biotechnological application.

## ANNOUNCEMENT

At the time this manuscript was prepared, only two Geotoga species were known, G. petraea and G. subterranea (1, 2). Members of the genus *Geotoga* were isolated from oil deposits and are anaerobes fermenting carbohydrates and proteinaceous compounds, are capable of reducing elemental sulfur to hydrogen sulfide, and cause pipeline corrosion and oil souring (1). The genome of the *G. petraea* type strain is presently unavailable, and no data exist on the intraspecies phenotypic diversity, which made it necessary to investigate the new *G. petraea* HO-Geo1 strain and to sequence its genome.

Strain HO-Geo1 (VKM B-3300) was isolated from the Vostochno-Anzirskoe petroleum reservoir (Russia) (3) by sequential transfers from the highest dilutions on RM liquid medium containing (in grams per liter) NaCl, 20.0; МgCl_2_ · 2H_2_O, 18.0; Na_2_SO_4_, 3.0; CaCl_2_ · 6H_2_O, 1.5; KCl, 0.5; NH_4_Cl, 0.33; NaHCO_3_, 0.2; yeast extract, 1.0; peptone, 5.0; ferric citrate, 0.02; and Na_2_S · 9H_2_O, 0.2 (pH 6.8 to 7.0) at 48°C. When examined on an Axio Imager D1 epifluorescence microscope (Carl Zeiss, Germany), the cells of strain HO-Geo1 were motile non-spore-forming rods with a sheath-like outer structure specific to members of the order *Thermotogales*. The strain was grown in the RM medium for 7 days at 48°C under anaerobic conditions. DNA was purified from the cell biomass using the cetyltrimethylammonium bromide (CTAB) method (4). The 16S rRNA gene was amplified with 27F and 1492R primers (5), and purified PCR products were sequenced with an ABI Prism 3730 DNA analyzer (Applied Biosystems, USA). The 16S rRNA sequence analysis using a BLASTn (6) search against the NCBI database revealed that HO-Geo1 shares 99.2% similarity with Geotoga petraea T5^T^ (GenBank accession no. NR_104910).

DNA libraries were prepared with the NEBNext DNA library prep kit for Illumina (New England BioLabs). Next-generation shotgun sequencing of the genomic DNA was carried out using the HiSeq 1500 platform (Illumina, Inc., USA). A total of 1,946,596 250-bp single-end reads were obtained from strain HO-Geo1. Low-quality reads were trimmed using Trimmomatic version 0.36 (7) with the default settings for single-end reads. Subsequently, the quality-filtered reads were *de novo* assembled with SPAdes version 3.12.0 using the default settings (8). The final draft genome assembly of HO-Geo1 contained 22 contigs, covering a total of 2,150,220 bp, with an *N*_50_ value of 331,226 bp, *L*_50_ of 2, G+C content of 29.38%, and average sequence coverage of 140×. Identification of the protein-coding sequences and primary annotation were performed using the NCBI Prokaryotic Genome Annotation Pipeline (PGAP; version 4.7) (9), which identified 2,058 genes, 1,997 protein-coding sequences, 9 pseudogenes, and 52 RNA genes.

Functional analysis performed with the Rapid Annotations using Subsystems Technology (RAST; version 2.0) server (10) via the RAST*tk* pipeline with the default settings (11) showed that 149 genes were associated with protein metabolism, 114 genes were associated with the metabolism of amino acids and their derivatives, 100 genes were associated with carbohydrate metabolism, and 49 genes were associated with the metabolism of cofactors, vitamins, prosthetic groups, and pigments.

The genome sequence provided here is expected to broaden our knowledge regarding the genetic and functional characteristics of the genus Geotoga.

### Data availability.

The GenBank/EMBL/DDBJ accession number for the 16S rRNA gene sequence of strain HO-Geo1 is MK984240. This whole-genome shotgun project was deposited at DDBJ/ENA/GenBank under the accession no. SRME00000000. The version described in this paper is version SRME01000000. The associated BioProject, BioSample, and SRA accession numbers are PRJNA530105, SAMN11295181, and SRR8846986, respectively.
